# Lipid-Based Drug Delivery Nanoplatforms for Colorectal Cancer Therapy

**DOI:** 10.3390/nano10071424

**Published:** 2020-07-21

**Authors:** Chunhua Yang, Didier Merlin

**Affiliations:** 1 Institute for Biomedical Sciences, Center for Diagnostics and Therapeutics, Digestive Disease Research Group, Georgia State University, Atlanta, GA 30302, USA; dmerlin@gsu.edu; 2 Atlanta Veterans Affairs Medical Center, Decatur, GA 30033, USA

**Keywords:** colorectal cancer, lipid-based nanoparticles, targeted drug delivery, plant-derived lipid nanoparticles, exosomes

## Abstract

Colorectal cancer (CRC) is a prevalent disease worldwide, and patients at late stages of CRC often suffer from a high mortality rate after surgery. Adjuvant chemotherapeutics (ACs) have been extensively developed to improve the survival rate of such patients, but conventionally formulated ACs inevitably distribute toxic chemotherapeutic drugs to healthy organs and thus often trigger severe side effects. CRC cells may also develop drug resistance following repeat dosing of conventional ACs, limiting their effectiveness. Given these limitations, researchers have sought to use targeted drug delivery systems (DDSs), specifically the nanotechnology-based DDSs, to deliver the ACs. As lipid-based nanoplatforms have shown the potential to improve the efficacy and safety of various cytotoxic drugs (such as paclitaxel and vincristine) in the clinical treatment of gastric cancer and leukemia, the preclinical progress of lipid-based nanoplatforms has attracted increasing interest. The lipid-based nanoplatforms might be the most promising DDSs to succeed in entering a clinical trial for CRC treatment. This review will briefly examine the history of preclinical research on lipid-based nanoplatforms, summarize the current progress, and discuss the challenges and prospects of using such approaches in the treatment of CRC.

## 1. Introduction

Colorectal cancer (CRC), a malignant neoplasm that starts in the colon or rectum, is one of the most common forms of gastroenteric cancer worldwide [1,2]. In the USA, nearly 150,000 new patients are diagnosed annually, and approximately 35% of them will die from CRC [3,4]. The cause of CRC is not fully understood; it is believed to be multifactorial, and accumulated evidence suggests that certain risk factors are linked to the disease [5]. For example, genetic factors (family history, inherited syndromes, racial and ethnic background), lifestyle (diet, smoking, and alcohol use), and other illness histories (inflammatory bowel diseases, colon polyps, obesity, type II diabetes) are reported to be strongly correlated with the formation of colon cancer [6,7,8,9,10,11,12,13].

**Figure 1:**
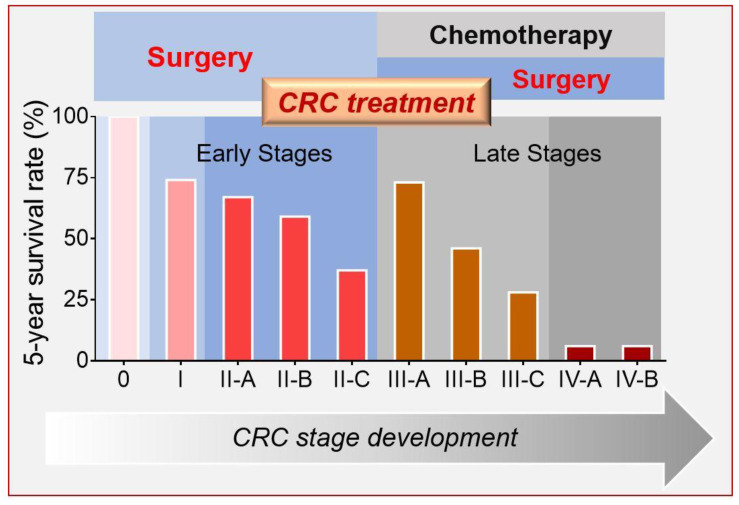
Colorectal cancer (CRC) stages and correlated clinical treatment.

CRC can be classified into five stages using the guidelines of the American Joint Committee on Cancer (AJCC) [14]. The earliest-stage CRCs are called stage 0 (a very early cancer); from there, the disease progresses through the early stages (I to II-C) to the late stages (III-A to IV-B) (Figure 1). Although the survival rate of early-stage CRC patients is relatively high (above 50%, stages 0 to II-B), those diagnosed in advanced stages (for example stages IIIB–IVB) exhibit an extremely low survival rate (as low as 5%) even after surgical resection [15,16]. For this reason, adjuvant chemotherapy (AC) is needed to improve the life expectancy of patients diagnosed in advanced stages [17,18,19,20].

Various ACs have been developed to slow tumor growth, block recurrence, or prevent metastasis [21,22]. These chemotherapeutic agents are highly efficient in vitro, but their nonspecific in vivo organ distributions often trigger a wide range of side effects in the clinic (including bone marrow toxicity, reproductive toxicity, hair loss, nausea, and vomiting) [21,23,24]. Physicians and scientists have designed sophisticated therapeutic protocols to minimize such side effects; these strategies often include complicated dosing intervals of chemotherapeutic agents and optimized drug-drug combinations [19,25,26,27]. However, the nonspecific bio-distribution of the chemotherapeutics remains a major barrier in efforts to reduce their toxicity [28,29,30,31]. The use of a colon-specific drug-delivery system (DDS), especially those based on nanotechnology-based colon drug delivery, might be a feasible solution for these problems.

Several types of nanoparticle-based DDSs (e.g., liposomes, natural-derived nanoparticles, biodegradable polymeric nanoparticles, and dendrimers) have recently been used to deliver therapeutics to the colon [32,33,34,35,36] successfully. Some functional nanoparticles can even target a specific sub-population of colon cells by employing cell-surface transporters or receptors, such as CD-98 [32], integrin [33], or folic acid (FA) receptors [34]. Further, nanoparticles that target metastatic CRC cells have emerged as a new strategy for CRC treatment [35]. As more than 50% of the late-stage CRC patients demonstrate a high risk of liver metastasis [36], many nanomedicines have been carefully designed with the goal of decreasing CRC-originated metastasis [37,38]. Liposomal nanoparticles were pioneered in nano-DDSs for cancer treatment because they offer superior safety and biocompatibility over other nanoparticles (including polymers and inorganic nanoparticles) [39]. In this review, we will describe the cutting-edge lipid-based nanotechnologies that have been developed for the treatment of CRC and metastatic CRC in preclinical studies. We will also discuss the challenges and prospects of such therapeutic strategies.

## 2. Current Lipid-Based Nanoplatforms

### 2.1. Liposomal Nanoparticles

**Figure 2:**
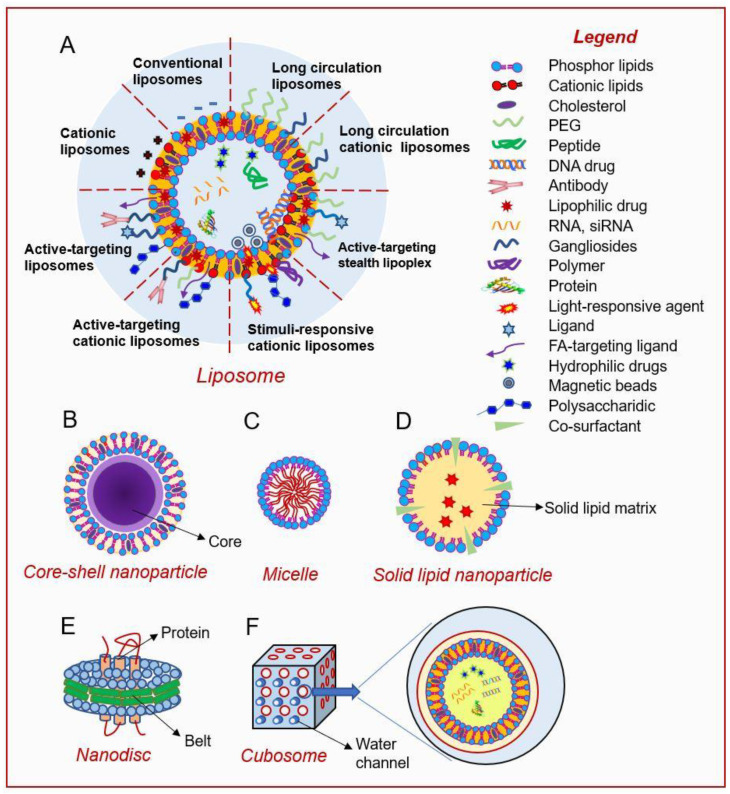
Lipid-based nanoplatforms for drug delivery: (**A**) liposomes, (**B**) core-shell nanoparticle, (**C**) micelle, (**D**) solid lipid nanoparticle, (**E**) nanodisc, and (**F**) cubosome.

Liposomes are nano- to micro-sized spherical lipid vesicles first described by A. Bangham in 1961 [40]. They have a cell-like morphology consisting of an internal aqueous core surrounded by an external phospholipid bilayer envelope [41] (Figure 2A). This unique structure allows liposomes to encapsulate and deliver both lipophilic and hydrophilic chemicals [42]. The structure of the liposome’s outer layer mimics those of biological membranes, and thus facilitates the easy incorporation of liposomes to cells via fusion or phagocytosis [43]. Because of its excellent biocompatibility and biodegradability, the liposome-based carrier is one of the safest DDSs; indeed, it was the earliest nanoplatform approved for clinical use by the US Food and Drug Administration (FDA) [44]. Pharmacologically, liposomal nanoparticles have shown the ability to significantly improve the pharmacokinetics and biodistributions of loaded drugs (especially lipophilic drugs) [45]. Such improvements naturally enhance the drug’s selectivity, offer prolonged drug release, and reduce the toxicity to healthy tissues. These advantages make liposomes particularly useful in cancer therapy. Today, liposomal DDSs are being extensively studied in various capacities, such as in the application of conventional chemical drugs [46,47], biomolecules [48,49], gene deliveries [50,51], and immune therapies [48,52].

#### 2.1.1. Conventional Liposomes

First-generation liposomes (conventional liposomes) are mainly composed of natural phospholipids (e.g., phosphatidylcholine [PC]), sphingolipids, and cholesterol [53]. As these compositions are commonly found in bilayer bio-membranes, conventional liposomes are highly biocompatible and nontoxic. In addition, a high drug-loading capacity can be achieved by adjusting their sizes and membrane compositions [53]. Despite these advantages, conventional liposomes proved unsatisfactory for clinical use: Their in vivo stability is problematic because they tend to fuse or self-aggregate, resulting in premature drug release and rapid systemic clearance by the mononuclear phagocyte system (MPS) [54].

#### 2.1.2. Long-Circulating Liposomes (LCLs)

The second generation of liposomes, comprising the so-called LCLs or stealth liposomes, was designed to overcome the limitations of conventional liposomes. LCLs incorporate hydrophilic-polymer-modified phospholipids in their outer layer, allowing them to avoid issues with self-fusion or self-aggregation and reducing their chances of being recognized by the MPS [55]. The most common phospholipid modification is polyethylene glycol (PEG) conjugation (or PEGylation) (Figure 2A). PEGylation is especially conducive to the reduction of the interaction between liposomes and plasma proteins, the improvement of liposome stability, and the enhancement of the mean residence time (MRC) of liposomes in circulation [56,57]. These improvements allow the chemotherapeutic drug to reach the tumor, where it is often delivered via the enhanced permeability and retention (EPR) effect [58]. The first well-characterized LCL was PEGylated liposomal doxorubicin (dox/PEG-L). The dox/PEG-L vesicles, which were 80 to 90 nm in diameter, had a circulation half-life of 2–3 days, which is hundreds of times longer and yielded a delivered drug concentration (in the tumor tissue) up to six times higher, compared to the results obtained using conventional doxorubicin [59].

The limitation of PEGylated stealth liposomes is that PEG modification inhibits the cellular uptake of the liposomes and often causes them to fail to escape from endosomal entrapment, potentially leading to significant loss of DDS function [60]. This critical problem associated with PEGylated liposomes, called the “PEG dilemma” [61], severely limits the ability of such liposomes to deliver low-dosage therapeutics, such as siRNAs or protein drugs.

Alternatively, the coating of liposomes with gangliosides (endogenous sialic acid-containing glycosphingolipids) can enable the liposomes to avoid immune recognition and thereby increase their MRC in circulation [62]. Some naturally derived hydrophilic polymers, such as chitosan, can also be used to decorate the surface of liposomes [63]. Chitosan is a hydrophilic biodegradable polymer that is widely regarded as nontoxic. As chitosan is positively charged, it can be easily coated on liposomes (which are negatively charged) via an ionic interaction [64]. The resulting alteration of the surface charge can be used to monitor the efficiency of chitosan coating. In an in vitro drug release assay, A. Alomrani et al. [65] revealed that chitosan-coated liposomes could more effectively reduce 5-fluorouracil leakage than conventional liposomes, and chitosan coating increased the cytotoxicity of 5-fluorouracil toward CRC cells (HT-29) in a sustained manner. These discoveries suggest that LCLs are promising DDSs with the potency of increasing the stability, safety, and efficacy of the delivered drug.

#### 2.1.3. Active-Targeting Liposomes

New generations of liposome-based DDSs have incorporated active-targeting nanotechnologies into their designs [66] (Figure 2A). The development of CRC-targeting nanoparticles has often been guided by advances in our understanding of CRC pathophysiology. In many cases, differences in cell-surface proteins (such as receptors and transporters) between CRC cells and healthy colorectal cells can be used to suggest potent drug-deliverable targets.

Following the discovery that urotensin-II receptor (UTR) is overexpressed by several epithelial cancers (including CRC and prostate cancer), S. Zappavigna et al. designed urotensin-II-conjugated liposomal nanoparticles (LipoUTs) [67]. LipoUTs exhibited a strong active-targeting capability for UTR-overexpressing CRC cells (such as WiDr and LoVo), which took up almost two times more LipoUTs than conventional liposomes. This design strongly enhanced the selectivity and efficiency of the drug carrier [67]. Folate receptor is another example of a cancer-overexpressed receptor that is often found on CRC cells [68]. S. Handali et al. and E. Moghimipour et al. both prepared FA-coated liposomes and loaded them with 5-fluorouracil. The resulting FA-nano-liposomes demonstrated excellent active-targeting capabilities and enhanced drug absorption by various types of CRC cells [34,69].

#### 2.1.4. Stimuli-Sensitive Liposomes

Cancer microenvironmental cues (such as pH gradient and reactive oxygen species [ROS] stress) [70,71] and stimuli (such as thermal energy, magnetism, and light) have been employed to design triggered-release liposomes [72,73,74] (Figure 2A). Liposomes designed to release their cargo in the intracellular cancer environment often have pH-sensitive polymers incorporated into their structure, as this enables the encapsulated drug to be released when the liposome reaches the acidic microenvironment [75]. For example, high-molecular-weight poly-(styrene-*co*-maleic acid) (SMA) exhibits a pH-related conformational transition, shifting from a charged extended structure (above its p*K*_1_) to an uncharged globule (below its p*K*_1_ value) [76,77]. This conformation change in the copolymer chain of SMA-liposomes destabilizes the liposomes at mildly acidic pH and releases the encapsulated cargo. S. Banerjee showed that SMA-liposomes exhibited excellent biocompatibility and stability, and could more efficiently deliver 5-fluorouracil into CRC cells (HT-29) than regular liposomes [76,77].

Alternatively, thermosensitive or photo-sensitive polymers can be included in the liposomal structure to enable controlled drug release in response to a temperature change or light of a specific wavelength [74,78]. Thermosensitive liposomes are often composed of 1,2-dipalmitoyl-sn-glycerol-3-phosphocholine (DPPC), which can facilitate cargo release when the microenvironment approaches its phase-transition temperature [79]. Nevertheless, the encapsulated drug is released relatively slowly from DPPC-liposomes, and application of the hyperthermia function often proves difficult. To improve the release kinetics, Needham et al. used lysolipids (such as MPPC or MSPC), lipid-grafted PEG, and DPPC to generate a liposomal doxorubicin formulation (dox/LTSLs) [72]. This formulation, commercially called ThermoDox^®^, can exhibit responses at lower temperatures than regular DPPC-liposomes; the drug is released upon a mild hyperthermic trigger and the system can deliver 25-fold more doxorubicin into tumors than intravenous (IV) delivery of the drug. A clinical study showed that ThermoDox^®^ is a promising nanoliposome for treating CRC liver metastases in combination with radiofrequency ablation [80,81].

#### 2.1.5. Cationic Liposomes

Cationic liposomes can be produced by two distinct methods: (A) incorporating positively charged lipids, such as 1,2-dioleoyl-3-trimethylammonium-propane (DOTAP), dioleoyl-phosphatidyl ethanolamine (DOPE), and dimethyl-dioctadecyl ammonium bromide (DODAB), on the surface of liposomes [82]; or (B) decorating conventional liposomes with cationic polymers, such as chitosan, atelocollagen, polyethyleneimine (PEI), cyclodextrin, and poly-L-lysine [83,84,85]. Alteration of the surface charge of a liposome can increase its ability to bind target cells and undergo uptake, as the addition of positively charged lipids will increase the interaction of liposomes with negatively charged cell surfaces [86].

Initially, cationic liposomes were developed for the delivery of anticancer agents. The first cationic liposomal formulation to undergo clinical trial was EndoTAG^TM^-1, a paclitaxel formulation that was designed for pancreatic cancer [87,88]. Interestingly, research showed that EndoTAG-1 could also stabilize the tumor for 4 weeks in patients with advanced CRC and liver metastasis [89]. As cationic liposomes often form stable liposome-nucleic acid complexes (named lipoplexes) with negatively charged nucleotides, cationic liposomes are expected to be a promising non-viral platform for delivering nucleic acid drugs for gene therapy [90] (Figure 2A). Compared to the viral nucleic acid delivery platform, cationic liposomes exhibit relatively low toxicity and are easy to produce and structurally simple (without immunogenic viral proteins) [91]. K.L. Lan et al. examined the inhibitory effect of cationic liposome-delivered murine endostatin gene (mEndo/Lipo) on the growth of intraperitoneally disseminated CRC tissue [92]. They found that although the efficiency of cationic liposome-mediated gene transfection was not better than that obtained using a viral system, the cationic liposome generated less immunogenicity, allowing the therapeutic genes to be delivered more frequently to the tested animals [92]. These findings suggested that the safety of cationic liposomes is critical for long-term tumor control by liposome-mediated gene treatments.

Cationic liposomes are the most common synthetic nano-platform for nucleic acid delivery currently used for cancer gene therapy [93,94]. A major challenge limiting the use of cationic lipids is that the constructed liposomes can extensively interact with negatively charged constituents in the circulation (such as serum proteins and opsonins), which may result in hemolysis [95]. Cationic liposomes may also activate the complement system and undergo rapid clearance by the macrophages of the reticuloendothelial system [96]. PEG coating has been widely used to enhance the systemic residence time of cationic liposomes and potentiate the systemic delivery of nucleic acids for gene silencing [58]. Stabilized nucleic acid-lipid particles (SNALPs), developed by Tekmira Pharmaceuticals, represent one such formulation [97,98]. The lipid bilayer of a SNALP comprises a mixture of cationic lipids, PC, cholesterol, and PEG-modified lipids. The PEG-modified lipids, which are presented on the outer layer, can shield and stabilize the SNALP and prevent it from being cleared from the system. Cationic lipids are accumulated on the inner layer, favoring the formation of siRNA/lipid complexes. SNALPs have a mean particle size of 100 nm and have been successfully investigated for systemic siRNA delivery [97,98].

### 2.2. Variants of Liposomes

#### 2.2.1. Prodrug Approach

The liposomal prodrug approach often employs lipid-drug conjugates (LDCs) to form a type of self-organized structure [99]. Most LDCs are amphiphilic compounds made by covalently conjugating the drug to the lipids through a linker. In a buffer solution, the synthesized amphiphilic LDCs can assemble themselves into nanoscale liposomal particles. The LDCs’ covalent bond or the linked lipid is susceptible to disease-related enzymes, such as secretory phospholipase A2. The self-assembled particles will thus attach to the desired target and then undergo activation and disassociation, leading to the precise release of the drug.

A. Arouri and O.G. Mouritsen investigated the hydrolytic profiles of two prodrug-containing liposomal formulations: (A) DPPC/prodrug binary mixtures (10–50 mol% prodrug), and (B) DPPC/prodrug/1,2-dipalmitoyl-sn-glycerol-3-phosphoethanolamine (DPPE)-PEG ternary mixtures (30 mol% prodrug, 5–10 mol% DPPE-PEG with average molecular weights of 350, 750, 2000, and 5000) [100]. As the linked DPPC is susceptible to phospholipase A2, the phospholipase hydrolysis rate of the prodrug was positively correlated with the percentage of DPPC. Surprisingly, the PEGylation also actively promoted the hydrolysis rate. These results indicated that the prodrug’s release behavior could be flexibly tuned by the addition of other liposome-forming components.

In some cases, non-lipophilic drugs have been conjugated with lipophilic components simply to increase their lipophilicity. The conjugated lipophilic prodrug was then added to the bilayer-forming lipids and assembled to be part of the lipid bilayer of the nanoparticles. J. Xing et al. designed a stable liposomal formulation containing a lipophilic prodrug of SN38 (moeixitecan), DPPC, hydrogenated soybean phosphatidylcholine (HSPC), and distearyl phosphatidylethanolamine (DSPE)-PEG2000 [99]. The moeixitecan-containing liposomes (moe/CLs) significantly improved the drug’s (SN38) solubility and stability and exhibited sustained drug release kinetics. Furthermore, the moe/CLs demonstrated significantly enhanced cytotoxicity and selective promotion of HT-29 cell apoptosis compared with irinotecan. In a colorectal xenograft model, moe/CLs showed better therapeutic efficacy than free moeixitecan and the clinically approved irinotecan [99].

#### 2.2.2. Core-Shell Lipid Nanoparticles

Core-shell lipid nanoparticles are often composed of a non-aqueous core (comprising different types of materials) and a lipid shell [101] (Figure 2B). This strategy can increase the drug-loading capability and the particles’ stability because such cores are designed to both contact the loaded drugs and support the outer lipid layer. Another benefit of the non-aqueous core is that its nanostructures may confer a controlled-release function [102]. Various cores, including metal nanoparticles, synthetic polymers, hydrogels, and micelles, have been explored for constructing the core-shell nanoparticles [101,103,104]. These lipid-based core-shell nanoplatforms can encapsulate different types of drugs and may render pharmacokinetic optimization. These nanoparticles also take full advantage of the benefits of conventional liposomes constructed with the same lipid outer membranes, including their biocompatibility and cell-uptake mechanism. In a study comparing the properties of pure liposomes and core-shell liposomes, the envelopment of metal-core nanoparticles inside the liposomes was found to have little influence on the liposomes’ properties. Once deposited on the cancer cells, the metal-core liposomes were taken up by the cells through clathrin-mediated endocytosis and immediately transferred to lysosomes; they thus exhibited a behavioral sequence identical to that of pure liposomes [105].

Among the various core-shell lipid nanoparticles, those with cores composed of poly-lactic-co-glycolic acid (PLGA) or poly-lactic acid (PLA) are among the most valuable systems for drug delivery [104]. They take advantage of both polymeric cores and liposomes: The polymeric core acts as a support that provides enhanced mechanical stability, controllable morphology, and a narrowed size variation; the PLGA or PLA polymers largely increase the surface-area-to-volume ratio of the cores to improve the controlled release ability of the nanoparticles; the lipid envelope enhances the biocompatibility, prolongs the circulation time, and prevents drug leakage; and the lipid-enveloped shell facilitates surface modification, including targeted modification and hydrophilic modification, which can be functionalized to maximize the delivery/co-delivery capability. To investigate the synergetic effects of such a formulation in a network of multiple non-apoptotic programmed cell death (PCD) pathways, X. Hou et al. prepared and characterized hyaluronic acid (HA)-modified, lipid-coated PLGA nanoparticles and loaded them with mRIP3-pDNA [106]. The HA-modified lipid core-shell nanoparticles exhibited excellent biocompatibility and high tumor-targeting efficiency. Impressively, the tumor inhibition rate was more than 80% in the CT26 mouse model when used in combination therapy with systemically delivered chloroquine. This effective CRC treatment was achieved by combining chemotherapeutics and core-shell delivered gene therapeutics to induce several interdependent PCD pathways [106].

### 2.3. Lipid Micelles

Lipid micelles are amphiphilic lipids that self-aggregate in a spherical form in aqueous solutions [107] (Figure 2C). Micelles have been extensively studied as a DDS for hydrophobic drugs, and recent major efforts have focused on improving the micelle’s drug loading capacity and in vivo stability, and reducing the micelle-cell interaction in circulation. PEG–lipid micelles (primarily conjugates of PEG and DSPE, or PEG–DSPE) have emerged as promising DDSs to address these limitations [108]. PEG–DSPE lipid micelles can be flexibly grafted and easily entrap therapeutics, placing them among the most versatile and practical carriers for cancer therapeutics. PEG–lipid micelles can target pathological sites through passive targeting (the EPR effect), or active targeting may be achieved by conjugating the PEG’s terminal group to a targeting ligand [109].

Y. Lu et al. investigated the siRNA delivery efficiency of a cationic PEG–lipid micelle [91]. The authors prepared the micelle by modifying a methoxy-poly(ethylene glycol)-poly(ε-caprolactone) copolymer (mPEG-PCL) micelle with a cationic lipid (DOTAP), and loaded the prepared micelle with siMcl1 or siBcl-xl. The resulting siMcl1/mPEG-PCL or siBcl-xl/mPEG-PCL micelle complexes were tested for their efficacy in a mouse CRC model [91]. Intra-tumoral injection of siMcl1/mPEG-PCL or siBcl-xl/mPEG-PCL micelle complexes effectively suppressed xenografted colon cancers in mice, suggesting that this modified lipid micelle could be a potent DDS for delivering gene therapeutics in the treatment of colon cancer.

The same authors subsequently reported a more complicated active-targeting micelle that was comprised of FA-conjugated mPEG-PCL and loaded with curcumin (Cur/FA-Mic) to target CRC cells with overexpressed FA receptor [109]. Cur/FA-Mic micelles had an average particle diameter of 30.47 nm. Compared to free curcumin, Cur/FA-Mic-delivered curcumin exhibited significantly improved pharmacokinetic constants, including an elongated half-life [T_1/2_] and notably increased drug exposure [109]. Not surprisingly, an in vivo efficacy study showed that Cur/FA-Mic exhibited strong effects in suppressing tumor growth, promoting tumor apoptosis, and attenuating angiogenesis to a much greater degree than free curcumin or empty micelles. These findings clearly demonstrate that FA-coated active-targeting micelles are potent platforms for delivering hydrophobic agents in colon cancer treatment.

### 2.4. Solid Lipid Nanoparticles (SLNs)

SLNs are colloidal lipid particles that possess a solid lipid core matrix at the physiological temperature [110] (Figure 2D). SLNs are generally made of lipids and surfactants and can be produced by different methods, including homogenization, ultra-sonication, micro-emulsion, and solvent emulsification/evaporation. SLNs are safer than other polymer-based nanoparticles because their non-payload lipids are generally nontoxic compounds [111,112].

Similar to micelles, SLNs have been considered to represent a very promising DDS for CRC therapy, particularly for the oral delivery of lipophilic anticancer drugs (e.g., doxorubicin and docetaxel) [113,114]. SLNs offer a larger drug-loading capacity, longer drug-retention time, and better overall particle stability than many other polymers, resulting in enhanced overall bioavailability of loaded drugs to the targeted tissue. Moreover, sustained or controlled drug release can be achieved by adjusting the degradation rate of the lipid matrix of SLNs.

To facilitate the sustained-drug release of docetaxel, K.S. Kim et al. constructed docetaxel-loaded cationic SLNs, which were further coated with an anionic polymer conjugated with glycocholic acid (Doc/SLNs-GA) [115]. Doc/SLNs-GA (~120 nm in diameter) actively targeted the distal ileum, and this was found to be mediated by the apical sodium bile acid transporter. After a single oral dose, Doc/SLNs-GA sustainably released docetaxel to the blood for up to 24 h, and such treatment did not show any sign of impairing the immune systems of treated mice. Daily oral administration of Doc/SLNs-GA inhibited the growth of existing tumors and prevented tumor formation. During treatment, the population of cytotoxic T cells increased while the population of tumor-associated macrophages and regulatory T cells decreased [115]. The SLN DDS allowed docetaxel to be given as a low-dose daily oral treatment, as the SLNs effectively reduce the systemic toxicity of the chemotherapeutic. Such a strategy could potentially help CRC patients maintain the therapeutic effects of docetaxel by using an intermittent low-dosage treatment between the maximally tolerated dose cycles. Importantly, this might prevent tumor recurrence and remission.

Although SLNs offer many advantages over other nano-DDSs, they retain the limitation of poorly encapsulating hydrophilic drugs, which distribute inadequately in the melted lipid droplets and show low drug-loading efficiency during SLN preparation [116,117]. To solve this problem, H.L. Wong et al. proposed a method of adding organic counterions during SLN preparation [117]. This process aimed to form ion pairs consisting of the charged hydrophilic drug molecule and the added organic counter ion, with the goal of increasing the distribution of the drug in the melting lipids. Another strategy is to form polymer-lipid hybrid nanoparticles, in which the hydrophilic drug is electrostatically attached to polymer counter ions and the drug-polymer complexes are distributed into lipid droplets for SLN preparation [118]. These two strategies improved the encapsulation efficiency of ionic drugs, such as by increasing the encapsulation of verapamil-HCl from 20 to 80% [117], and have thus greatly expanded the future application of SNLs.

### 2.5. Lipid Nanodiscs

Nanodiscs are disc-shaped nanoparticles (often <50 nm) that comprise a lipid membrane and a belt (made of a polymer or peptide) that holds the disc together [119] (Figure 2E). Nanodiscs offer great advantages in delivering membrane proteins, lipophilic drugs, or protein-drug combinations, which is especially useful for combinational therapy. In addition to maintaining the disc’s structure, the polymer belt can be modified to activate the targeting function of the nanodisc.

Recently, R. Kuai et al. constructed a novel high-density lipoprotein (HDL)-mimicking nanodisc and loaded it with doxorubicin [120]. The nanodisc potentiated the immune checkpoint blockade in a murine CRC model. Delivery of doxorubicin via HDL-nanodiscs triggered the immunogenic cell death of CRC cells, exerting excellent antitumor efficacy without any overt side effects caused by off-target effects. The administration of nanodiscs elicited robust antitumor CD8^+^ T cell responses, broadening T cell epitope recognition to tumor-associated antigens, neoantigens, and intact tumor cells. Combination therapy (nanodiscs and anti-programmed death-1) induced complete regression of established CT26 and MC38 CRC tumors in more than 80% of the tested animals. No tumor recurrence was observed in the surviving animals [120]. This work provided strong evidence that nanodisc-based chemotherapy is capable of initiating antitumor immunity and sensitizing tumors to immune checkpoint blockade.

### 2.6. Nano-Cubosomes

Cubosomes are liquid crystalline particles that are formed from the lipid cubic phase and stabilized by a polymer outer corona [121] (Figure 2F). The lipid cubic phase of the cubosome is formed by a self-assembly process in which amphiphilic lipids are mixed with water at a proper ratio. The cubosomes share a unique architecture comprising a continuous lipid bilayer and curved water channels [122]; this facilitates the enclosure of a variety of drugs with hydrophilic or lipophilic properties. The pore sizes of cubosomes can be adjusted by modifying the lipid composition, and the outer corona of the cubosomes can be engineered for a targeting function.

Nano-cubosomes, which are made by disintegrating the large cubic-phase particles, have nanostructures that are nearly identical to those of cubosomes with a significantly higher membrane surface-area-to-volume ratio [123]. This gives the nano-cubosomes a stronger membrane protein-binding ability than similar-sized liposomes. Nano-cubosomes also have a much lower viscosity than regular cubosomes and are usually highly stable under physiological conditions [124]. These unique properties make nano-cubosome an excellent potential delivery platform for oral drug delivery.

M.M. Saber et al. used an emulsification technique to construct nano-cubosomes bearing cisplatin (cis/Cub) or a cisplatin-metformin combination (cis/met/Cub) [125]. The constructed cis/Cub exhibited superior cytotoxic effects against CRC cells (HCT-116) compared to free cisplatin [125]. Interestingly, the cis/met/Cub nanoparticles were found to induce CRC cell apoptosis by disturbing several metabolic pathways (e.g., via AMPK activation and mTOR inhibition), depleting glucose, and reducing the energy level. The application of cis/met/Cub also suppressed p-Akt (Ser473) and escalated the ROS concentration, thereby increasing nicotinamide-adenine-dinucleotide-phosphate (NADPH) oxidase, decreasing lactate-dehydrogenase (LDH), and considerably enhancing caspase-3 activity. Together, these mechanistic studies provide the foundation for the further development of effective cubosome-based combinational therapy to treat colon cancer.

### 2.7. Plant-Derived Lipid Nanoparticles (PDLNPs)

**Figure 3:**
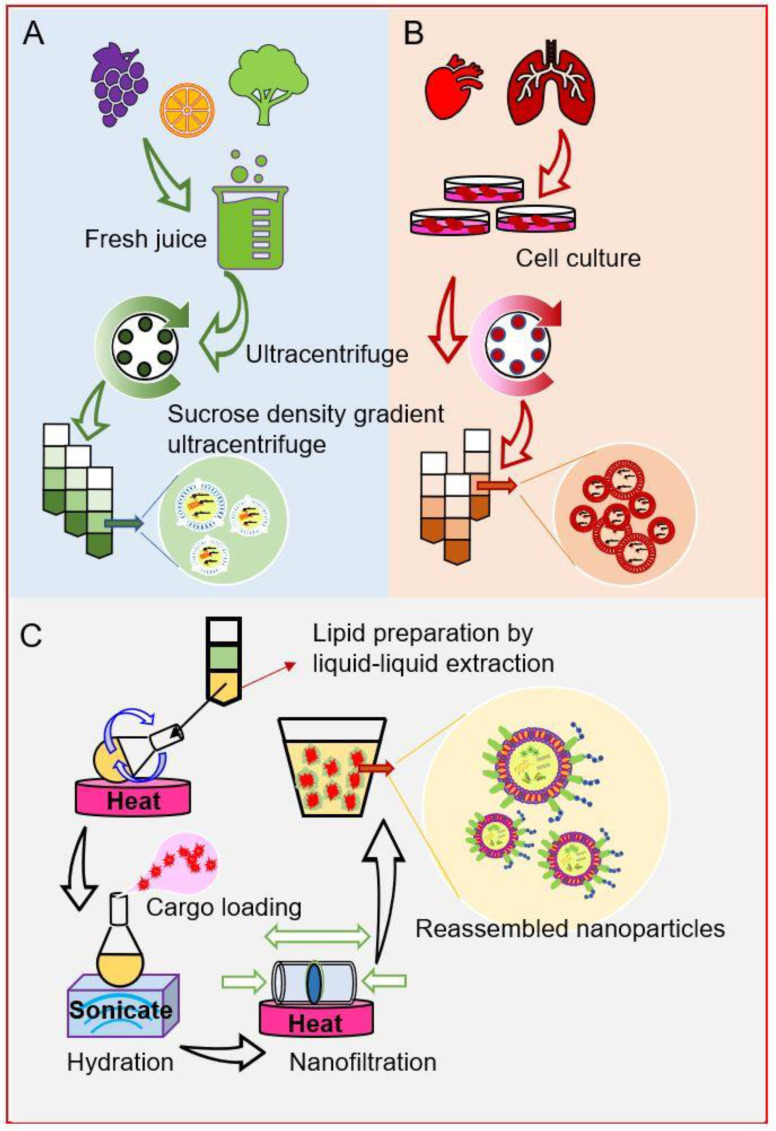
Natural occurring nanoparticles for drug delivery. (**A**) Isolation of plant-derived nanoparticles, (**B**) preparation of mammalian cell-derived exosomes, (**C**) engineering natural-derived nanoparticles from extracted lipids.

Researchers have recently characterized a series of naturally occurring nanoparticles derived from fresh vegetables or fruits, including broccoli, garlic, ginger, grape, grapefruit, and lemon [38,126,127]. Upon oral administration, native PDLNPs can efficiently target specific tissues, such as the colon (ginger-derived lipid nanoparticles) and liver (grapefruit-derived lipid nanoparticles) [38,128,129]. Transmission electron microscopy (TEM) revealed that these native PDLNPs are spherical nano-sized particles that share a liposome-like structure, with an aqueous core and lipid bilayer [130] (Figure 3A). Unlike the lipid bilayers of liposomes, those of PDLNPs are full of glycolipids (such as monogalactosyl-diacylglycerol [MGDG] and digalactosyl-diacylglycerol [DGDG]) and phospholipids (PA, PI, and PE), but lack cholesterol [126]. As the primary function of cholesterol in an artificial liposome is to stabilize the lipid bilayer, the lack of cholesterol is commonly believed to potentially compromise the stability of PDLNPs. However, Q. Wang et al. discovered that grapefruit-derived nanoparticles are more stable than cationic liposomes when both were incubated with 10% bovine serum at 37 °C [131]. These authors also showed that grapefruit-derived nanoparticles maintained their structures in a 4˚C buffer solution for more than 30 days. In a similar study, M. Zhang et al. found that ginger-derived nanoparticles were stable for more than 4 weeks in the stomach- and intestine-like solutions and were also tolerant of multiple freeze/thaw cycles [130].

Interestingly, native PDLNPs can have anti-CRC benefits. For example, M. Zhang et al. found that a sub-fraction of native ginger-derived nanoparticles (GDNPs-2) prevented chronic colitis and colitis-associated colon cancer [130]. These anticancer effects may be attributed to the actions of encapsulated ginger natural products, such as small-molecule secondary metabolites, proteins, peptides, and miRNAs [130,132]. This suggests that native PDLNPs have the potential to deliver their own cargos to modulate damaging factors and promote healing in the colon region.

Further studies revealed that the liposome-like structure of PDLNPs could be reproduced by self-assembly when total lipids extracted from PDLNPs were hydrated in a buffer solution [126,133] (Figure 3C). This self-assembly ability of PDLNP-extracted lipids offered a novel drug delivery strategy [133,134]. M. Zhang et al. found that IV injection of reassembled ginger-extracted-lipid-derived nanoparticles with FA-coated surfaces could deliver doxorubicin to the CRC tumor site [129]. These reassembled nanoparticles showed significantly increased targeting efficiency and were able to reduce the tumor volumes in mice xenografted with colon cancer (colon-26) cells.

### 2.8. Exosomes from Mammalian Cells

Exosomes are extracellular nano-sized vesicles (around 30 to 100 nm) that are secreted by most mammalian cells [135,136]. The lipid compositions may differ drastically between plant-derived nanoparticles and animal cell-derived exosomes, but they share a similar liposomal structure and cell-specific targeting functions [126] (Figure 3B). Exosomes are extraordinarily biocompatible, given that they participate in routine cell-cell communication and continuously shuttle between the host and receptor cells [136]. Furthermore, as these exosomes have specific innate targeting ability, they can be employed to construct highly specific nano-delivery systems.

G. Liang et al. constructed 5-fluorouracil- and miR-21 inhibitor oligonucleotide (miR-21i)-encapsulating exosomes using engineered 293T cells [137]. The application of these exosomes significantly down-regulated miR-21 expression in the 5-fluorouracil-resistant HCT-116^5FR^ cell line. The co-delivery of miR-21i and 5-fluorouracil by the exosomes significantly boosted the cytotoxicity of 5-fluorouracil and effectively reversed the drug resistance in these 5-fluorouracil-resistant CRC cells. Further, systematic administration of 5-fluorouracil- and miR-21i-loaded exosomes in tumor-bearing mice yielded a very potent antitumor effect [137]. These studies demonstrated the high capability of exosomes as a potential targeted DDS.

## 3. Deliverable Targets for CRC Treatment

### 3.1. Receptors and Transporters on CRC Cells

Most cancerous cells reproduce far more frequently than normal cells and accordingly overexpress specific types of replication-promoting receptors and transporters on their surfaces [138]. Conjugating ligands (such as vitamins, peptides, or hormones) or incorporating specific antibodies of these receptors or transporters onto the surface of a liposome could, therefore, be an efficient active-targeting strategy [69,139,140]. For example, FA receptors and transporters are overexpressed in many types of cancers, including CRC. Therefore FA-coated liposomes are expected to exhibit increased uptake by cancer cells with overexpressed folate receptors [141,142]. E. Moghimipour et al. showed that, in CT26 cells, FA-decorated liposomal 5-fluorouracil exhibited enhanced cellular uptake of the encapsulated 5-fluorouracil compared to free 5-fluorouracil, with a three-fold decrease in the IC_50_ (12.02 versus 39.81 µM, respectively) and 25-fold higher ROS production (62,271.28 versus 2369.55 a.u., respectively, as shown by fluorescence intensity) [69]. In an animal study, this FA-decorated liposomal formulation showed significantly greater tumor growth inhibition compared to free 5-fluorouracil (tumor volume, 88.75 versus 210.00 mm^3^, respectively). The excellent biocompatibility and safety of this FA receptor-targeting strategy were verified by hemolytic assays and histological examinations. In a similar study, S. Handali et al. confirmed that the in vitro cytotoxicity (tested by MTT assay) of an FA-coated liposomal 5-fluorouracil formulation was much higher than those of conventional liposomes and free 5-fluorouracil against various CRC cell lines (e.g., HT29, Caco2, and CT26 cells) [34,142]. The targeted liposomes were also found to induce necrosis in vitro in HT29 cells.

Studies on human colonic biopsy specimens indicated that the expression levels of amino acid or peptide transporters, such as CD98 and PepT1, were significantly increased in patients with CRC [32,143], suggesting that these transporters might be potential therapeutic targets for drug delivery. This hypothesis was validated by using CD98 antigen-binding fragment (Fab′)-functionalized nanoparticles to co-deliver a CD98 siRNA (siCD98) and the anticancer drug, camptothecin, to CRC cells [32]. CD98-guided nanoparticle delivery produced much better anti-CRC and anti-migration effects compared to other relevant nanoparticles. Although the siCD98 was delivered by non-lipid polymeric nanoparticles, the study still suggested that this amino acid transporter might be an efficient delivery target for the treatment of CRC.

### 3.2. Target the Colon Cancer Stem Cells (CCSCs)

Accumulating evidence suggests that the elimination of all cancerous colon cells, especially CCSCs, is critical to eradicating CRC [144]. Otherwise, relapses and/or metastases are highly likely in CRC patients [145,146]. To enable researchers to better understand the role of CCSCs in CRC relapse and metastases, we need to develop adequate techniques for isolating and identifying CCSCs [146,147]. Such studies will be conducive to the development of CCSC-specific therapies. Currently, feasible strategies include delivering anti-cancer drugs directly to CCSCs and increasing the sensitivity of CCSCs to available chemotherapeutics [35,148]. Unlike regular cancer cells, CCSCs appear to replicate slowly; thus, they can escape formulations that target quickly replicating cells. Fortunately, some populations of CCSCs can be characterized by their specifically expressed biomarkers, such as CD133, CD44, CD166, EpCAM, and Lgr5 [149,150,151,152]. Targeting one or more of these biomarkers could offer a practical approach for effectively delivering anticancer drugs to CCSCs. For example, the CCSC biomarker, CD44, is the receptor of hyaluronic acid (HA), which is a natural polysaccharide found in normal human bodies. Nanoparticles with HA decorated surfaces may have the ability to target CD44-expressing CCSCs [153]. B. Mansoori et al. developed an HA-modified liposome formulation that encapsulated 5-FU (5-FU-lipo-HA) with an average size of 144 ± 77 nm [75]. When this formulation was applied to a CD44-expressing CRC cell line (HT29) versus a non-CD44 expressing hepatoma cell line, the results revealed that 5-FU-lipo-HA exhibited optimal cell uptake and release of 5-FU into HT29 cells, but only minimal cell uptake of 5-fluorouracil into the hepatoma cells [75,154]. This HA-modified liposome formulation efficiently triggered cell-cycle arrest and cell apoptosis, and noticeably decreased the colony formation of CRC cells.

### 3.3. CRC Microenvironment

The CRC microenvironment is the complex environment surrounding the heterogeneous population of CRC cells. It includes the extracellular matrix, stromal cells, immune cells, secreted factors, signaling molecules, and surrounding blood vessels [155,156,157]. This microenvironment plays a vital role in CRC progression, and the colon cancer itself reversely tunes the microenvironment to favor its proliferation. Signaling molecules such as ROS can act as important microenvironmental indicators and regulators for immune responses, cell proliferation, and tissue damage in different stages of CRC development. In the later stages of CRC growth, CRC-secreted factors promote angiogenesis (the generation of new blood vessels) such that the extension of blood vessels into the CRC mass supports tumor growth and facilitates metastasis.

Given that cancer cells manage to coexist with immune cells through a combination of camouflage and reconfiguration of the microenvironment, nanoparticles that potentially interrupt the microenvironment may offer an approach to elicit an antitumor immunity that can be useful for cancer treatment [158,159]. In recent research, X. Duan et al. used lipid-based core-shell polymer nanoparticles to effectively deliver an immunostimulatory chemotherapeutic combination that altered the microenvironment of CRC [160]. The delivered chemotherapeutic combination (oxaliplatin and dihydroartemisinin) exhibited strong synergy in generating ROS. The elevated ROS effectively activated immune responses and synergized with an anti-PD-L1 antibody to treat CRC in a murine model. The use of lipid-based core-shell polymer nanoparticles significantly improved the biodistribution and tumor uptake of the chemotherapeutic drugs. More importantly, this nanoplatform eliminated the risk for peripheral neuropathy associated with oxaliplatin and activated the innate and adaptive immune systems, and thus elicited durable and long-lasting antitumor immunity [160,161]. Under repeated dosing, tumor recurrence was prevented and all tumors were eventually eradicated. Although this work is in an early stage, the authors predict that this biodegradable and well-tolerated immunostimulatory treatment will prove effective in clinical trials against CRC.

In another study, T. Yu et al. employed a liposomal anti-angiogenesis drug (apatinib mesylate liposomes, or Lip-Apa) in a combinational therapy for CRC [162]. Co-administered Lipo-Apa and liposomal docetaxel (DOC/M) showed strong synergistic effects, inhibiting cell proliferation and inducing the apoptosis of CT26 cells in vitro. In a subcutaneous xenograft CRC model, a combination of Lipo-Apa (gavaged) and DOC/M (locally delivered) significantly decreased angiogenesis, CRC proliferation, and abdominal metastasis [162]. This study suggested that the targeting of microenvironmental factors that favor angiogenesis could have synergetic effects with chemotherapies, and may have the potential for clinical treatment of CRC.

### 3.4. Target the CRC Metastatic Liver Cancer

Metastatic CRC is a major cause of cancer relapse and cancer-related death in the late stage of CRC (stage IV), and the liver is the leading site of CRC metastasis [35]. In the late stage of CRC, circulating CRC cells enter the liver microvasculature via the hepatic portal vein. Given that the microcirculation slows down at the extensive branching of the portal vessels, which connects to the hepatic sinusoids [163], the circulating CRC cells are mechanically retained in the liver, making this organ extremely susceptible to CRC metastasis.

The liver filters the bloodstream and is a major metabolic organ. Thus, most intravascularly delivered lipid nanoparticles accumulate in the liver tissues, assuming that they exhibit sufficient circulation time and a suitable size [164]. The surface modification of conventional liposomes by polymerization, such as PEGylation, can significantly increase the circulation time of liposomes. U. Pohlen et al. showed that hepatic arterial-infused 5-FU/PEGylated-liposomes exhibited improved anti-metastatic efficacy with long-lasting tumor regression and prolonged survival times in rats [165]. In a tissue bio-distribution study, H. Ichihara et al. prepared hybrid liposomes (HLs) with polyoxyethylene dodecyl ether and L-α-dimyristoyl phosphatidylcholine (DMPC), and labeled the HLs with a fluorescent dye [166]. The authors observed that the fluorescent-labeled HLs accumulated in the liver tissues of xenograft mouse models of CRC liver metastases for more than 24 h after their IV injection. This demonstrated the potential value of LCLs in treating CRC liver metastases.

Another group used more specific liver-targeting polysaccharides to decorate the surfaces of the liposomes. Galactose-coated liposomes were found to exhibit a stronger specific cell uptake by human hepatocellular carcinoma HepG2 cells when compared to non-targeted liposomes [47]. The antitumor effects of liposomal formulations with or without a galactose coating were evaluated using a murine model of CRC with liver metastasis. The results indicated that the progression of tumors in the liver and mesenteric lymph nodes was significantly suppressed by doxorubicin/galactosylated-liposomes administered via spleen injection, whereas non-galactosylated formulations did not have any significant effect. These data indicated that local perfusion of galactosylated liposomal doxorubicin holds great promise for the treatment of liver metastasis from CRC [47]. In another study, HA was exploited for a liver-targeting function [167]. T. Jiang et al. developed dual-functional liposomes with HA and pH-sensitive cell-penetrating peptide (CPP) to target encapsulated paclitaxel to the liver. Functionalized liposomal particles accumulated at the tumor site through passive (EPR effect) and active (HA-guided) targeting; thereafter the HA outer corona was detached by HAase, the pH-responsive CPP was exposed, and the cellular uptake of liposomes was enhanced by the cell-penetrating effect [167]. The penetrating ability produced endosomal/lysosomal escape for efficient intracellular paclitaxel delivery, which had an evidently higher tumor-inhibitive effect than paclitaxel in in vitro and in vivo studies.

### 3.5. Lipid Nanoparticles Mediated Gene Therapy

CRC gene therapy involves the introduction of nucleic acids (deoxyribonucleic acid [DNA] or ribonucleic acid [RNA]) into disease-related cells [82,168,169]. This strategy may employ two approaches: (A) correcting the defective gene(s) by silencing/deleting cancer genes or activating the anticancer genes; or (B) activating the immune system to stimulate an immune response capable of recognizing and eliminating CRC cells. Although gene therapy has enormous potential for cancer patients with advanced or recurrent CRC, major obstacles remain, such as poor targeting selectivity of the delivery systems and inefficient gene transfer [170,171]. Generally, the success of the treatment depends on the carrier’s ability to precisely and efficiently deliver the nucleic acids to the desired targets without affecting healthy cells.

Historically, the most popular way to deliver genes into human cells was to use deactivated viruses as the delivery system (so-called viral gene delivery) [172,173]. Such methods are still used on occasion. However, viral gene delivery requires stringent manufacturing conditions because a trace amount of impurities from the deactivated virus (such as viral proteins or RNAs) may cause dangerous adverse immunogenic reactions. Therefore, in the past decade, non-viral gene delivery methods have gradually replaced the viral gene delivery method [86]. Today, lipid and polymer-based non-viral gene delivery carriers dominate the field of gene therapy; they offer many advantages, including ease of manufacturing, excellent biocompatibility (safety), and flexibility for design [170].

The liposome was the first type of non-viral nano-platform exploited for its nucleic acid delivery ability [174]. As nucleic acids are negatively charged, cationic lipids (e.g., DOTAP and DOPE) are incorporated into the liposomes to change the surface charges of both the inner and outer layers to net positive values. The positively charged liposomes interact with the nucleic acids via electrostatic attraction to form a relatively stable complex (lipoplex). The lipoplex enters the cell through endocytosis and may incorporate itself into the endocytic membrane transport pathway [175]. Once the lipids fuse with the cell organelle membrane, nucleic acids are released from the lipoplex. However, the stability of the lipoplex can be compromised by interactions between the liposomal outer layer and various in vivo factors, limiting the potential clinical use of such “cationic” liposomes. A number of techniques have been developed to increase the stability and selectivity of the lipoplex by changing the surface charge of the outer layer and adding cell-specific targeting functions. For example, T. Wang et al. constructed cationic-LCLs by combining distearoyl-phosphatidylcholine (DSPC), cholesterol, dioctadecyl-dimethylammonium chloride (DODAC), and N-palmitoyl-sphingosine-1-succinyl (PEG-CerC16) at a 25/45/25/2.5 molar ratio [50]. The constructed nanoliposomes effectively encapsulate the survivin siRNA: DODAC bound to the survivin siRNA to form a lipoplex, and the PEG-modified lipid outer layer increased the residence time in circulation. The siRNA/cationic-LCLs significantly reduced the expression level of survivin and inhibited the cell proliferation of CRC cells both in vitro and in vivo [50]. This study verified that lipid nanoparticles could be a potent vector for delivering nuclear acids to treat colon cancer.

The delivery of engineered antigens may also activate the immune system and yield an excellent therapeutic effect. Surface modifying liposomes with antigens and encapsulating the antigens inside the liposomes can elicit the desired immunologic response [52]. For instance, H. Guan et al. reported that the human mucin-1 peptide (BP-25) could elicit a potent specific T-cell response when subcutaneously delivered either in an encapsulated liposomal form or attached to the surface of liposomes [176]. Interestingly, modifying the surface charge of the liposomes can also affect the immunogenicity of the particles. Manipulation of the constituents of the lipid bilayer of cationic liposomes can alter the surface charge density and eventually induce immune responses. K.S. Kim et al. constructed a docetaxel cationic SLN by coating the liposome with an anionic polymer conjugated with glycocholic acid (DSLN-CSG, ~120 nm in diameter) [115]. DSLN-CSG was actively absorbed in the distal ileum via interactions with the apical sodium bile acid transporter. In mouse models, oral administration of DSLN-CSG inhibited the growth or prevented the formation of colorectal adenocarcinoma. The treatment increased the population of cytotoxic T cells but reduced the populations of tumor-associated macrophages and regulatory T cells [115]. This study suggested that low-dose daily oral treatment may help prevent tumor recurrence in patients through immune regulations triggered by lipid-based nanomedicines.

### 3.6. Multiple Targeting Strategies

Cancer is typically considered a heterogenic disease or a combination of multiple diseases, and CRC is no exception [177,178]. Accumulating studies have shown that a combination of multiple drugs, sometimes called cocktail therapy, is necessary for the treatment of CRC.

FOLFOX, a combinational therapy composed of folinic acid (FnA), 5-fluorouracil, and oxaliplatin (OxP), has been used to treat CRC for many years [179]. However, conventional FOLFOX treatment requires a long period of time and suffers from high toxicity. J. Guo et al. thus developed a new formulation to address these limitations [180]. The authors used an active form of OxP ([P_t_(DACH)(H_2_O)_2_]^2+^) and FnA to create a nanoprecipitate (C_26_H_35_N_9_O_7_P_t_), and then encapsulated it in a PEGylated lipid nanoparticle (aminoethyl anisamide-targeted). The generated Nano-Folox demonstrated excellent multiple-targeting functions and optimized pharmacokinetics. It provided anticancer effects and induced immunogenic apoptosis (chemo-immunotherapeutic activities) in the orthotopic CRC mouse model. Further, the combination of Nano-Folox and 5-fluorouracil presented significantly stronger chemo-immunotherapeutic responses than FOLFOX without toxicity [180]. In combination with an anti-PD-L1 monoclonal antibody, Nano-Folox/5-Fu decreased liver metastases in mice. These results suggested that the Nano-Folox-based combination strategy is potent for the treatment of CRC.

In another example, V. Juang et al. employed a combined strategy composed of two lipid-based nanoparticles: a pH-sensitive peptide-modified liposome (PML) loaded with the chemotherapeutic drug, irinotecan, and SLN-encapsulated miR-200 [70]. These PMLs included one ligand (targeting tumor angiogenesis) and two types of peptides with distinct functions (one cell-penetrating peptide and one mitochondria-targeting peptide). The PMLs were further decorated with pH-sensitive PEG-lipid derivatives via an imine bond. The resulting nanoparticles exhibited excellent pH-responsive release and immediate internalization/intracellular distribution in CRC (HCT116) cells, with low toxicity to blood and healthy intestinal cells. Co-delivery of miR-200 loaded SLN further increased the cytotoxicity of irinotecan-pH-sensitive PMLs against CRC cells. Animal studies further indicated that these multi-targeting functionalized nanoparticles exhibited supreme therapeutic outcomes by inhibiting CRC growth and reducing systemic toxicity. Mechanistically, this combination was found to suppress the R.A.S./β-catenin/ZEB multiple drug resistance (MDR) pathways and induce CRC cell apoptosis [70]. Overall, these data suggest that the use of multifunctional nanoparticles to deliver a combination of miR and chemotherapy may offer a potential regimen for effectively treating CRC.

## 4. Delivery Routes of Lipid-Based Nanoparticles in CRC Treatment

**Figure 4:**
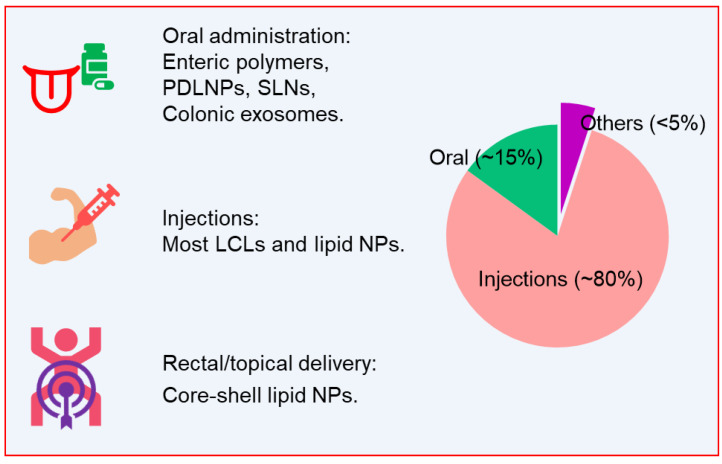
Currently developed delivery routes of lipid-based nanoparticles for CRC treatment.

The site-specific delivery of therapeutics to the colon would have multiple benefits for treating CRC, such as increasing the effectiveness, reducing systemic side effects, and lowering the dosage [66,181]. Such benefits would also facilitate the strategy of low-dosage long-term treatment because the toxicity and nonspecific distribution of the drug can be largely reduced. There are many routes for delivering therapeutics to the colon, including oral administration, IV delivery, injection, and other less-popular ways (e.g., rectal delivery) [182,183] (Figure 4). In many cases, non-gastrointestinal cancers have been treated by therapeutics delivered via the IV approach [184,185]. IV delivery can yield high systemic bioavailability by bypassing the liver’s first-pass effect. However, CRC treatment can be amenable to oral administration as long as the DDS can protect the loaded drug and release it in the colon [186].

### 4.1. Oral Administration

Oral administration is obviously the most favorable delivery route, as CRC patients can easily follow drug compliance in the treatment cycle [187]. The major challenge for orally delivering lipid-based nanoparticles relates to the environmental extremes found in the gastrointestinal (GI) tract. Various factors, including pH, transit time, enzymes, and microbiota, may affect the stability and targeting efficiency of orally administered nanoparticles [66,188]. However, these factors may also offer enormous opportunities for designing orally deliverable lipid-based nanoparticles.

For example, colonic pH plays a vital role in regulating aspects of colonic hemostasis (e.g., colonic cell growth, control of absorption and secretion, and degradation of bile acids) [189], and consistent observation of altered colonic pH values may indicate the incidence of CRC [190]. In a retrospective study, D. Charalambides and I. Segal compared the colonic pH between CRC patients and healthy persons and reported that the fecal pH tended to be higher in CRC patients (pH 6.6) than normal conditions (pH 6.0) [189]. Based on the distinctive pH values of the respective GI tract segments, lipid-based nanoparticles can be designed to deliver drugs directly into the colon. K. Rajpoot and S.K. Jain introduced FA-grafted SLNs bearing irinotecan, which was encapsulated in the microbeads of alginates that were coated with Eudragit^®^ S100, a type of pH-responsive enteric polymer [191,192,193]. These SLNs/microbeads were designed to release the drug only into the intestinal region (with pH > 7.0). Their colon-specific targeting ability was confirmed in an optimized radiolabeled biodistribution study, and the microbeads showed a residence time of Insert up to 48 h inside the colon cancer tissue. These SLNs/microbeads exhibited a significantly (*p* < 0.01) higher antitumor effect (against HT-29-derived cancer) than control beads in an animal study [191]. Together, these data suggest that orally deliverable SLNs/microbeads show enhanced anti-CRC efficacy.

The composition and abundance of microbiota in the colonic region vary remarkably between colitis-associated colon cancer patients and healthy individuals [194]. Thus, microbiota species-sensitive lipid nanoparticles could be constructed for a colon-targeted DDS [128]. Similarly, other distinctive physiological characteristics of the GI tract (e.g., ligand-receptor pairs, enzymes, colonic transition time, and pressure) could also be employed for designing oral DDSs for treating CRC.

Lipid-based nanoparticle drug delivery strategies that seek to use a singular-targeting mechanism to treat CRC have shown little success in clinical studies [39,44]. Singular-targeting design lacks flexibility, and the nanoparticle may lose its selectivity when facing the complex and harsh GI environment. Thus, multiple-responsive nanoparticles have gained popularity in lipid-based DDS development [70]. Indeed, the successful SLNs/microbeads described above included a dual-responsive design: the Eudragit^®^ S100 coating of the microbeads facilitated the pH-targeted delivery, while the FA-grafted SLNs had a cancer-cell-targeting function. Going forward, multiple-responsive drug delivery is expected to take the lead in the treatment of CRC.

It is worth mentioning that naturally occurring nanoparticles, such as plant-derived nanoparticles (PDNPs) and mammalian cell-derived exosomes, have intrinsic multiple-responsive functions. Native PDNPs present excellent colon-targeting ability, potentially due to the unique compositions of their lipid bilayers, which have high contents of glycolipids and transmembrane proteins. For instance, M. Zhang et al. showed that ginger-derived nanoparticles (GDNPs) are composed of glycolipids (MGDG and DGDG), transmembrane proteins, ginger miRNAs, and ginger secondary metabolites (gingerols and shogaols). Orally delivered GDNPs were found to efficiently target the colon cells with a multiple-responsive ability that is likely to involve size-, ligand-, and receptor-mediated processes [130]. Interestingly, lipids extracted from GDNPs showed potent self-organizing properties, indicating the GDNP lipid-based nanoparticles can be engineered to have multiple functions. The same research group extracted the total lipids from GDNPs, constructed FA-coated nanoparticles, and successfully loaded them with doxorubicin [129]. A subsequent in vitro study showed that Dox/FA-NPs were efficiently taken up by CRC cells with no apparent toxicity; in contrast, cationic liposome controls presented strong signs of toxicity (decreased cell proliferation and increased apoptosis) at the same concentrations. This uniquely engineered Dox/FA-NP showed pH-dependent drug-release profiles and targeting of the FA receptor on the surface of colon-26 tumors in an in vivo study. Such dual-function-engineered PDNPs enhanced the chemotherapeutic effect of doxorubicin against CRC growth compared with free doxorubicin.

It is commonly believed that most mammalian cell-derived nanoparticles are not suitable for oral administration, as these exosomes are not stable when traveling along the GI tract. However, recent research showed that colonic exosomes might remain functional against the colonic disease after oral administration, indicating that colonic exosomes can maintain their structure (at least partially) in the GI tract and thus can be engineered as orally deliverable nanotherapeutics to treat CRC [136].

### 4.2. Injections and IV Delivery

Injectable routes, including IV delivery and intradermal [ID], intramuscular [IM], and subcutaneous [SC] injections, are the most efficient forms of drug delivery in terms of maximizing the drug’s systemic bioavailability [195]. However, high systemic bioavailability does not necessarily translate to a high local drug concentration and it inevitably increases the chances of systemic side effects in CRC treatment. Therefore, a highly specific targeting function must be included in the design of such nanoplatforms. Due to stability issues, most biologics (cancer vaccines, siRNAs, DNAs, and protein/peptide drugs) are delivered with the aid of long-circulating lipid-based nanoparticles via IV route or injections [196,197]. The passive-targeting approach (EPR effect) and active-targeting approaches (e.g., cancer microenvironment-dependent release) are often combined to optimize the efficacy of the drugs [198].

S. Tummala et al. found that tumor necrosis factor-related apoptosis-inducing ligand (TRAIL) is a promising new target for CRC treatment [199]. The authors formulated hybrid liposomal nanoparticles to co-deliver oxaliplatin and anti-TRAIL for treating CRC in xenograft tumor models. Oxaliplatin-loaded liposomes were coated with a polymeric chitosan layer and conjugated with an antibody (thiolated) to generate DSPE-PEG-mal3400. The chitosan-coated lipid layer prevented the drugs (oxaliplatin and anti-TRAIL) from being degraded in serum after IV delivery, while the conjugated anti-TRAIL specifically guided the nanoparticles to the CRC target. The effective delivery of both drugs by this strategy was evidenced by significant reductions in the tumor mass and tumor volume compared to the non-coated control nanoparticle-treated group [199]. These findings indicate that the active targeting approach of the antibody-decorated chitosan-coated liposomes could be a favorable approach for injectable CRC-targeting therapy. Today, various synthetic polymer-based nanoparticles are being examined in preclinical studies for IV drug delivery against CRC. However, most of them have not yet been sufficiently assessed in terms of their long-term safety. Only a few polymers, such as polyethylene glycol [PEG], PLGA, and chitosan, have been verified as being biocompatible [104,200]. Thus, the use of lipid-based nanoparticles alone or hybridized with verified polymers could be a safer approach for IV drug delivery.

Unlike the synthetic polymer-based nanoparticles, mammalian cell-derived exosomes (MCDEs) offer a very biocompatible system [137]. MCDEs continuously shuttle between host and receptor cells, playing essential roles in cell-cell communication and CRC progression [201]. For this reason, MCDEs have been widely studied for their potency in CRC diagnosis. With the development of new techniques for exosome isolation and preparation, some researchers have recently started using enriched or engineered exosomes as DDSs to treat CRC. For example, P. Tran et al. prepared CRC cell-derived exosomes and loaded them with aspirin to generate nano-amorphous aspirin-loaded exosomes [202]. The authors observed significantly improved cytotoxicity of aspirin and enhanced apoptosis and autophagy of CRC cells using this exosome formulation. Importantly, these engineered nano-amorphous exosomes were found to be capable of eradicating the cancer stem cells. A further animal study proved that aspirin-loaded exosomes efficiently delivered aspirin to xenograft tumors after IV injection [202]. An active-targeting function was added to these exosomes by the conjugation of an aptamer specifically targeting the EpCAM protein on CRC cells. This nano-amorphous structured exosomal DDS effectively transformed aspirin into a valid CRC therapeutic with the potential to eliminate the cancer stem cells. In another example, T. Liu et al. used miR-128-3p-transfected cells to package miR-128-3p into secreted exosomes [203]. The authors discovered that intratumoral injection of these miR-128-3p-loaded exosomes could effectively deliver miR-128-3p to oxaliplatin-resistant CRC cells. Such exosome-aided miRNA delivery largely improved the oxaliplatin response of oxaliplatin-resistant CRC cells and significantly reduced tumor growth, suggesting a new treatment strategy for oxaliplatin-resistant CRC patients.

In addition to exhibiting excellent efficiency, exosomes appear to be a safe injectable platform. G. Liang et al. evaluated the systematic toxicity of engineered exosome (THLG-EXO) in healthy BALB/c mice [137]. After IV injection of THLG-EXO every other day for 7 days (20 mg/kg), no death or severe body weight loss was observed and the tested biochemical parameters, including markers of liver function (alanine aminotransferase [ALT] and aspartate aminotransferase [AST]) and kidney function (creatinine [CRE] and blood urea nitrogen [BUN]) remained in a normal range similar to that of PBS-treated animals. These results indicated that THLG-EXO had no apparent hepatic or renal toxicity within the tested dosage regimen. Hematologic parameters, including the white blood cell (WBC), red blood cell (RBC), and platelet counts, also showed no significant difference between the THLG-EXO- and PBS-treated groups. Furthermore, no apparent histopathological abnormality or lesion was observed in major organs collected from the THLG-EXO-treated group [137]. Together, these results showed that multiple dosages of THLG-EXO could be safely delivered in an injectable form.

### 4.3. Other Delivery Routes

Accumulating evidence indicates that the rectal delivery of nanomedicines offers particular advantages for treating colonic diseases [182,204]. Such advantages include immediate therapeutic effects, diminished hepatic first-pass effects, and reduced pain experiences in patients. Although rectal delivery is recommended by many experts, it is less popular than oral administration or IV injection. Despite this, some preclinical researches have yielded exciting results. In studying the rectal delivery capability of a lipid-based nanosystem, L. Wang developed core-shell nanoparticles with a PEI/p21-saRNA-322 polyplex core and hyaluronan-coated lipid shell for the treatment of CRC [205]. These lipid-based core-shell nanoparticles were maintained at the rectum for more than 6 h and preferentially accumulated at the CRC tumor site. The core-shell nanoparticles exhibited excellent cellular update, and a CD44-knockdown assay indicated that this could be attributed to HA-CD44 recognition. In an orthotopic microsurgery mouse model of bioluminescent human CRC, these nanoparticles demonstrated superior antitumor efficacy [205]. These results provided preclinical proof-of-concept for a novel method to treat CRC by rectal administration of lipid-based nanoparticle-formulated p21-saRNA-322.

In general, the selection of the delivery route used to treat CRC depends on the individual’s disease stage and the properties of the selected drugs. When CRC is diagnosed in the early stages and the cancer is restricted in the colon, the oral and/or rectal routes would be a wise solution for delivering chemotherapeutics while reducing the systemic bio-distribution of the toxic drugs. Injectable administration of drugs is particularly useful for the delivery of biologics for short-term treatment of CRC patients of all disease stages. As biologics often exhibit a short in vivo half-life and notable instability, lipid-based nanoparticles are needed to escort the biologics in a stealthy and specifically targeted manner. For late-stage CRC patients, where the chances of metastasis are increased, injectable treatment is also a good option. However, the selection of injectable administrations for late-stage CRC treatment needs to balance many factors, including systemic toxicity, the biodegradability of nanoparticles, dual-targeting of CRC/metastasized tissue, targeting of cancer stem cells, and the alteration of the cancer microenvironment.

## 5. Conclusions and Prospects

Over the last 25 years, the development of lipid-based nano delivery strategies for cancer treatment has yielded substantial advances in both preclinical and clinical studies [39,44]. The first-generation lipid-based anticancer nanoparticles delivered loaded drugs to the tumor site through the passive EPR effect (offered by the vascular and lymphatic drainage of tumor tissues). Surface modification of the lipids extended the targeting functions of the nanoparticles, generating active-targeting and stimulus-responsive nanoparticles based on various targeting mechanisms. The ultimate goal of such modification is to give the nano-therapeutics precise cancer-targeting functions that can optimize the pharmacokinetics and bio-distribution of encapsulated drugs while minimizing their systemic side effects.

Several lipid-based nanoplatforms have become success stories in the medical field, such as Lipusu^®^ (paclitaxel liposome) and Marqibo^®^ (vincristine sulfate liposome) for the clinical treatment of gastric cancer and leukemia [206,207,208]. However, there is still a dearth of effective nanomedicine for clinical CRC treatment. Lipid-based nanoparticles of diverse shapes, nanostructures, and chemical natures represent one of the safest nanoplatforms and can encapsulate different types of anticancer cargos, including chemotherapeutics, peptides/proteins, siRNA, and DNAs (Table 1). It is not unreasonable to predict that these lipid-based nanoplatforms, especially LCLs (including cationic LCLs), will be the first type of nanoplatform to succeed in a clinical trial for CRC treatment.

CRC can be viewed as a highly heterogenic disease or can be recognized as a combination of different diseases [138,209], as different genetic and biological alterations are seen in different patients. Even within a single patient, there is high variability between the tumors at different development stages. Although the repertory of biomarkers identified from CRC, CCSC, and metastatic cancer cells can be exploited as targets for advanced nanoparticles, the treatment strategy (such as delivery route, dosage, dose frequency, and duration of cycles) depends on the needs and general state of the individual. It is worth to mention that, excessive fluctuations of the blood drug concentrations between chemotherapeutic cycles can promote the drug resistance from cancer cells, which may cause the failure of treatment and can noticeably decrease the survival rate of CRC patients. Such a complex scenario makes it extremely challenging to select the treatment strategy and design the relevant lipid-based DDS.

Scientifically, personalized nanomedicine is perfectly suited for treating diseases like cancer, as it will take into account individual differences in genetics, lifestyle, and living environment. Accordingly, there is an increasing need for a versatile nano-DDS that is compatible with such precise treatment. However, most nanocarriers are limited in terms of their flexible drug loading capacity and targeting selectivity. Lipid-based nanoparticles are the most advantageous choice as they offer a wide variety of particle sizes, adjustable dosage, sustained/controlled drug release, and versatile targeting functions. With advances in our understanding of genetics, cancer immunology, and cancer pathophysiology, lipid-based nanoparticles can be tailored to deliver personalized gene therapy, immune therapy, or combinational therapies to eradicate colon cancer in a simple treatment cycle.

## Figures and Tables

**Table 1:** Lipid-based nano-DSSs for CRC therapy.

Cargo	Formulation	Target	Category	Route	Stage	Ref.
5-fluorouracil	Chitosan-coated liposomes	HT29 cells	LCLs	N/A	Characterization	[65]
Doxorubicin	Urotensin-II-conjugated liposomes	WiDr or LoVo cells	Active targeting	N/A	Characterization	[67]
5-fluorouracil	FA-coated liposomes	HT29, CT26, and Caco2 cells	Active targeting	Subcutaneous injection	Preclinical	[34,69,142]
5-fluorouracil	SMA-liposomes	HT29 cells	pH-sensitive	N/A	Characterization	[76,77]
Doxorubicin	ThermoDox^®^	CRC metastatic liver cancer	Thermo-sensitive	IV infusion	Clinical trial	[80]
Paclitaxel	Cationic liposomes (EndoTAG-1)	Advanced CRC, liver metastasis	Cationic liposomes	IV injection	Clinical trial	[89]
Endostatin gene	Cationic liposomes	CT26 and HCT116 cells	Cationic liposomes	Intraperitoneal injection	Preclinical	[92]
PLK1 siRNA	SNALPs	PLK1 overexpressed CRC	LCL (PEG-modified) cationic liposomes	IV injection	Preclinical	[98]
Retinoids	DPPC or DPPC/DPPE-PEG	HT29 and Colon205 cells	Prodrug	N/A	Characterization	[100]
Moeixitecan	DPPC/HSPC/DSPE-PEG	HT29 cells	Prodrug	Subcutaneous injection	Preclinical	[99]
mRIP3-pDNA	HA-PLGA	CT26 cells	Core-shell, active targeting	IV injection	Preclinical	[106]
siRNA siMcl1/siBcl-xl	DOTAP/PEG-PCL	C26 CRC cells	Cationic micelle, gene therapy	Intratumorally injection	Preclinical	[91]
Curcumin	FA-conjugated mPEG-PCL	CT26 cells	Self-Assembly Micelles	IV injection	Preclinical	[109]
Docetaxel	Cationic SLNs	Distal ileum, CT26 cells	SLNs	Oral	Preclinical	[115]
Doxorubicin	HDL mimics	CT26, MC38 cells	Nanodisc	IV injection	Preclinical	[120]
Cisplatin, metformin	Pluronic-F127, glyceryl monooleate, polyvinyl alcohol	HCT116 cells	Nano-cubosome	N/A	Characterization	[125]
Doxorubicin	FA-coated Ginger LNPs	Colon26 cells	PDLNP, Active targeting	IV injection	Preclinical	[129]
6-shogaol	Ginger LNPs	Colon	PDLNP	Oral	Preclinical	[133,134]
5-fluorouracil, miR-21i	Engineered exosome	5-fluorouracil resistant HCT116 cells	Exosome, Multiple-targeting	IV injection	Preclinical	[137]
5-fluorouracil	HA-modified liposomes	HT29 cells	Active targeting (CD44)	N/A	Characterization	[75]
5-fluorouracil	PEGylated liposomes	CRC metastatic liver cancer	LCLs	Hepatic arterial infusion	Preclinical	[165]
Fluorescent dye	Mixture of vesicular and micellar molecules	HCT116, metastatic liver cancer	Hybrid liposomes	IV injection	Characterization	[166]
Paclitaxel	HA, cell-penetrating peptide	CRC metastatic liver cancer	pH-sensitive, cell-penetrating	IV injection	Preclinical	[167]
Survivin siRNA	Lipoplex	LoVo cells	Cationic LCLs, Gene therapy	Intraperitoneal injection	Preclinical	[50]
Mucin-1 peptide (BP-25)	Cationic liposome	T-cell	Immune therapy, Cationic liposomes	Subcutaneous	Preclinical	[176]
Docetaxel	Cationic SLN	Apical sodium bile acid transporter, T-cell	Immune therapy, SLN	Lymphatic transport	Preclinical	[115]
Folinic acid, 5-fluorouracil, oxaliplatin, anti-PD-L1 antibody	PEGylated lipid nanoparticle	CT26-FL3, metastatic liver cancer	Multiple-targeting, Immune therapy	IV injection	Preclinical	[180]
Irinotecan, miR-200	Peptide-modified liposome, SLN	HCT116, CT26 cells	Multiple-targeting (pH-sensitive, cell-penetrating, mitochondria-targeting)	IV injection	Preclinical	[70]
Oxaliplatin, anti-TRAIL	Immunohybrid liposomes	HT29 cells	Multiple-targeting	IV injection	Preclinical	[199]
Aspirin	CRC cell derived exosomes	HT29, CRC stem cells	Active targeting	IV injection	Preclinical	[202]
miR-128-3p	Exosomes	Oxaliplatin-resistant HCT116OxR cells	Gene therapy	Intratumorally injection	Preclinical	[203]
p21-saRNA-322	HA-lipid shell nanoparticles	HT29	Gene therapy, core-shell	Rectal delivery	Preclinical	[205]

N/A means “not available”.
